# Mineralocorticoid receptor antagonists promote renal immunosenescence

**DOI:** 10.1007/s11255-025-04530-1

**Published:** 2025-04-30

**Authors:** Qiao-Rui Wang, Yi Yang

**Affiliations:** 1 Department of Nephrology, Center for Regeneration and Aging Medicine, the Fourth Affiliated Hospital of School of Medicine, International Institutes of Medicine, Zhejiang University, Yiwu, Zhejiang China; 2 Zhejiang-Denmark Joint Laboratory of Regeneration and Aging Medicine, Yiwu, Zhejiang China

**Keywords:** Mineralocorticoid receptor, Finerenone, Kidney, Immunosenescence

## Abstract

Chronic kidney disease (CKD) is often associated with chronic inflammation, influenced by the activation of mineralocorticoid receptors (MR). This review focuses on changes in immune cells and explores the important role that MR antagonists (MRAs), especially the new nonsteroidal MRA, finerenone, play in alleviating renal and cardiac injury by affecting the transformation of stimulated immune cells. We found that MR can promote the transformation of macrophages to M1 pro-inflammatory phenotype through IL-14 receptor and mitogen-activated protein kinase (MAPK)–JNK. MR also activates helper T cells and reduces the generation of regulatory T cells by promoting the interaction between nuclear factor and activator protein-1, increasing the secretion of IL-2 and IL-18, increasing the expression of CD38 and CD69, especially the IL-17/IL-23 axis. The above immune system changes jointly mediate inflammation leading to kidney damage and fibrosis. In addition, we propose that the NLRP3 inflammasome is associated with macrophage imbalance. Preclinical studies indicate that finerenone effectively reduces inflammation and prevents structural kidney damage without significant systemic blood pressure changes. These data will provide some ideas for further research in the field of immune mechanisms in future, and drug research targeting specific targets and channels may also become a new type of diagnostic and treatment measure.

## Background

Patients with chronic kidney disease exhibit a coexisting inflammatory state and immune dysregulation. It has been demonstrated that kidney disease may result in the local enhancement of renal innate immunity, while simultaneously leading to the systemic activation and dysfunction of the innate immune system [1]. The process of aging is associated with alterations in the immune system, a phenomenon known as immunosenescence [2]. Aging reduces glomerular filtration rate (GFR) and impairs kidney function. In chronic kidney disease (CKD), aging contributes to structural damage and immune dysfunction. Combined with genetic/environmental factors that disrupt metabolism and increase oxidative stress, these age-related changes raise CKD risk in older adults [3].

Aldosterone binds to intracytoplasmic receptors, forming a hormone-receptor complex, which then enters the nucleus through the nuclear membrane. There, it synthesizes aldosterone-induced protein through various mechanisms. Inflammation has been shown to play an important role in the pathophysiology of chronic kidney disease [4]. Aldosterone can induce inflammation through pathological activation of mineralocorticoid receptors (MRs) in the kidney and heart [5].The mineralocorticoid receptor, a member of the nuclear receptor family, binds mineralocorticoids and glucocorticoids with high affinity [6], and is the major receptor for the hormone aldosterone. Previous studies have found that mineralocorticoid receptor activation has specific effects on various cell types – cardiomyocytes, endothelial cells, vascular smooth muscle cells, adipocytes, and inflammatory cells [7, 8]. Inflammation or immune system activation plays an important role in the pathophysiology of aldosterone-promoted vascular injury [9]. The renal protective effect of aldosterone blockade has been attributed to its antifibrotic activity, and the presence of inflammation is crucial in the pathogenesis of renal fibrosis [10]. The immune response ages with age and is characterized by increased susceptibility and a sustained systemic inflammatory response [3]. There is substantial evidence to suggest that MR antagonists possess an aldosterone-independent mechanism of action [6].This review examines the principal mechanisms of action of mineralocorticoid receptor antagonists (MRAs), including novel nonsteroidal MRAs, on renal immune cell regulation, and presents a synthesis of the clinical effects of MRAs.

## Expression and function of MR in renal immune cells

Inflammatory cells (dendritic cells, macrophages, and T cells) are all involved in aldosterone-modulated mineralocorticoid receptor-induced inflammatory responses in both renal and cardiac tissues. A substantial body of evidence from numerous studies indicates that the immune system plays a pivotal role in aldosterone-mediated fibrosis and tissue damage. Furthermore, these studies demonstrate that the immune system serves as a crucial mediator of MR.

### Expression and function of MR in macrophages

Macrophages play a role in inflammatory responses and the maintenance of tissue repair and remodeling. There is compelling evidence that the degree of infiltration of pro-inflammatory macrophages is closely correlated with the severity of renal injury in both acute kidney injury and chronic kidney disease models. IL-4 receptor is a key signaling pathway for the induction of M2 macrophages. MR activation interferes with IL-4 signaling and promotes the polarization of macrophages toward M2 pro-inflammatory phenotype [11]. The stimulation of steroid hormones in mouse macrophages has been observed to give rise to pro-inflammatory effects, manifested as a transformation into the M1 phenotype [12]. The activation of nuclear hormone receptors (PPARγ and PPARδ) directly drives macrophages toward a selectively activated state, namely, an M2 phenotype [13, 14].

MR deficiency has been demonstrated to suppresses migration and proliferation of macrophages and suppresses macrophage inflammatory responses via the SGK1-AP1/NF-κB pathway [15]. It seems that mineralocorticoid receptor signaling may stimulate the mitogen-activated protein kinase (MAPK)- cJun N-terminal kinases (JNK) pathway independently of other pathways[16]. The phosphorylation of JNK was markedly elevated in bone marrow-derived macrophages following exposure to LPS, a prototypical type 1 pro-inflammatory mediator.

In the presence of aldosterone, macrophages undergo rapid proliferation and infiltration. Infiltrating macrophages have the capacity to release a number of active products commonly associated with inflammatory responses, including lysosomal enzymes, nitric oxide, reactive oxygen intermediates, and TGF-β. These have been identified as key factors in the development of glomerular and tubular damage. In the event of damage to the glomeruli or renal tubules, a series of inflammatory factors and chemokines are produced, including chemokine-1 and RANTES (regulated by normal T cell expression and secretion). These factors can attract macrophages to proliferate and infiltrate the kidneys, migrate to the site of kidney damage, and subsequently release a substantial number of pro-inflammatory and oxidative stress products, thereby producing a phenomenon analogous to a cytokine storm.

NLRP3 inflammasome is a protein complex composed of NLRP3 protein, ASC, and caspase-1 [17]. It has been demonstrated that this process can facilitate the processing of caspase-1-mediated pro-inflammatory cytokines, namely, IL-1β and IL-18. In the kidneys of rats treated with aldosterone, there was an increase in the infiltration of macrophages and T lymphocytes, which were capable of producing a substantial quantity of inflammatory mediators, including MCP-1, TNF-α, inflammasome activation markers IL-1β, caspase-1, and NLRP3. Additionally, the formation of reactive oxygen species, as well as the IGF-1 signaling pathway, was observed [12, 18]. Aldosterone is an important activator of NLRP3 inflammasome, and its activation mechanism involves NF-κB signaling pathway. And the activation of the NLRP3 inflammasome is associated with an imbalance in the M1/M2 macrophage populations. Wu et al. demonstrated that the VEGFR3-AMPK-dependent autophagy pathway in macrophages can inhibit the activation of NLRP3 inflammasome in salt-sensitive hypertensive mice and alleviate NLRP3 inflammasome-associated CASP-1 activation in macrophages, thereby preventing renal inflammation [19].

### Expression and function of MR in T cell

It is evident that MR in T cell plays a critical role in the development of renal fibrosis [20]. Aldosterone further activates T cells through dendritic cell-dependent activation and secretion. Aldosterone can promote the interaction with the nuclear factor of activated T cells (NFAT-1) and activator protein-1 (AP1) by activating MR [21], thereby promoting the increase and infiltration of T cells and promoting the transformation of CD4 + T cells into pro-inflammatory T helper cells phenotype, as well as reducing T regulatory (Treg) cell number, which controls IFN-γ in T cells and regulates target organ damage [21].

The induction of aldosterone results in the simultaneous upregulation of CD69 and the secretion of IL-2 and IFN-γ, which in turn promotes increased activation of CD8 + T cells [22]. Thales et al. demonstrated that the blockade of the MR was capable of counteracting the thymic dysfunction induced by ethanol consumption. This was achieved by preventing the activation of nicotinamide adenine dinucleotide phosphate oxidase and the subsequent production of superoxide, which resulted in alterations to T cell maturation [23, 24]. MR plays a role in regulating peripheral T cells, promoting tissue infiltration and facilitating the activation of double-positive CD4⁺ and single-positive CD8⁺ cells [25]. In studies of rats treated with DOCA-salt, DOCA was found to activate helper T cells and reduce the production of regulatory T cells [26].

MR activation promotes T cell proliferation and differentiation, leading to the release of IL-17. The study revealed that IL-17 in mice is contingent upon IL-6 and TGF-β, which are produced by dendritic cells in response to aldosterone via the p38 and JNK MAPK pathways, polarizing CD4 T cells to a Th17 phenotype. Th17 cells require IL-23 for expansion and survival. IL-23 is released by activated dendritic cells and macrophages [27]. And γδ T cells, which, in addition to inducing the secretion of substantial quantities of IL-17, are also capable of secreting cytokines such as interferon γ, IL-6, and perforin [28]. In a DOCA-salt hypertensive mouse model, finerenone prevented cardiorenal injury by reducing renal accumulation of IL-17-producing RORγ⁺ γδ T cells, thereby lowering urinary albumin-to-creatinine ratio (UACR) and demonstrating renoprotection [29].

**Fig. 1:**
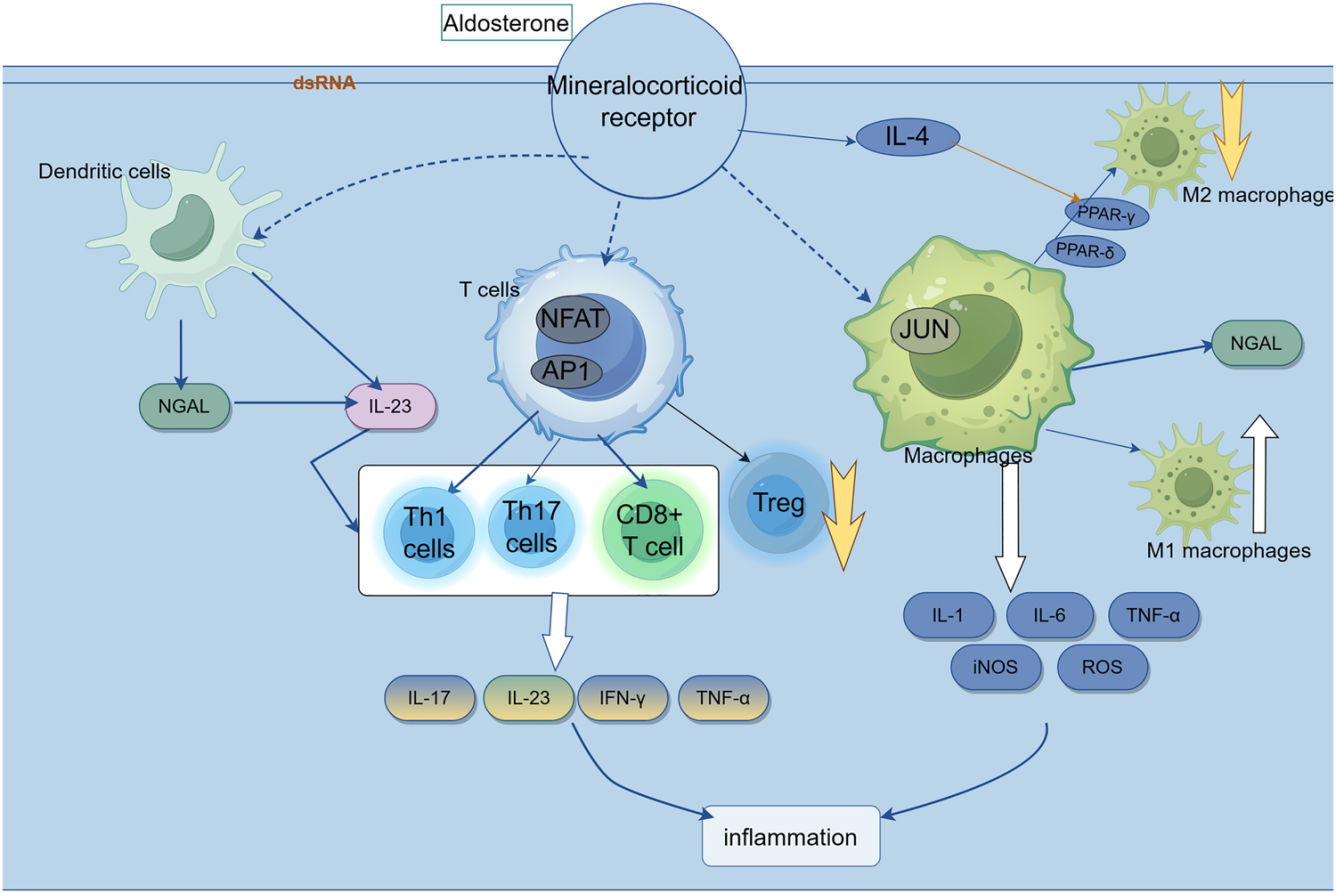
Aldosterone-mediated pro-inflammatory effects in macrophages and T cells. (By Fig-draw)

In MRKO mice, Ang II-induced glomerular hypertrophy was prevented and proteinuria levels were significantly improved. CD8 + cells exert a more pronounced influence on Ang II-induced hypertension and organ damage than CD4 + , and activated CD8 T cells secrete a greater quantity of IFNγ than CD4 T cells [30, 31]. (The above mechanisms are summarized Fig. 1**.**)

Under aldosterone stimulation, mineralocorticoid receptors in macrophages activate signal transduction of the MAPK-JUN pathway, promoting the transformation of macrophages to the M1 pro-inflammatory type. In T cells, MR activation promotes the action of nuclear factor of T cells (NFAT) and AP1, thereby stimulating the expression of helper T cells 1 (Th1) and helper T cells 17 (Th1), while reducing the number of regulatory T cells (Treg). In addition, it can promote the activation of CD8 + T cells. These processes are regulated by NGAL and IL-23 factors released by dendritic cells. The above multiple pathways work together to produce inflammatory mediators and cause tissue damage.

Renal insufficiency and hypertension exert a mutual influence upon one another [32]. Overexpression of the MR gene in T cells resulted in an elevation of blood pressure in mice treated with Ang II. Furthermore, the treatment of DOCA-salt rats with anti-IL-17 led to a notable reduction in arterial hypertension and the expression of profibrotic and pro-inflammatory mediators in the heart and kidneys, in addition to a reduction in collagen deposition [22, 26]

Serine/threonine protein inhibitor 1 (SKG1) is expressed in both T cells and dendritic cells [33]. SKG1 was shown to be a key player in salt-stimulated and Ang II-stimulated hypertension, as well as in the development of end-stage hypertensive damage [34, 35]. SGK1 plays a regulatory role in sodium transport in renal epithelial cells, upregulating the sodium-chloride cotransporter (NCC) and the sodium epithelial channel (ENaC) [36]. And SGK1 has the capacity to directly activate T cells, independent of the involvement of dendritic cells. The specific deletion of SKG1 in T cells has been demonstrated to alleviate salt-induced renal and vascular damage [37]. In comparison to the control mice, the absence of SGK1 in T cells was observed to result in the absence of any accumulation of memory cells or inflammatory cytokines, such as IFN-γ, in the kidneys [38].

### Neutrophil gelatinase-associated lipocalin

Neutrophil gelatinase-associated lipocalin (NGAL) has been shown to be a specific target for MR. There is a positive correlation between NGAL and mineralocorticoid receptor expression in immune cells, particularly in dendritic cells and macrophages [39]. The production of NGAL by immune cells is of great importance in the context of aldosterone-induced vascular fibrosis. And it has been demonstrated that NGAL is a necessary factor in aldosterone-induced inflammation in dendritic cells. In addition, Benjamin et al. demonstrated that in mice overexposed to mineralocorticoids, NGAL produced by macrophages plays a key role in renal fibrosis through the CCL5/IL-4 pathway [40].

### MR activation leads to increased expression of inflammatory factors

Oxidative stress has been demonstrated to promote the production of inflammatory factors and the activation of inflammatory cells. This is achieved by forming reactive oxygen species, which have the capacity to activate MR [41]. In proximal tubular cells, Ang II induces the expression of nuclear factor kappa B (NF-κB) [42], which in turn leads to the production of IL-6, TNF-α, and IFN-γ. These effects may be mediated by increased levels of peroxisome proliferator-activated receptor γ (PPAR-γ) protein and phosphorylated Smad protein, a reduction in the expression of cardiotrophin (CT-1), and an increase in Toll-like receptor 4 (TLR4) signaling [43–46]. The aforementioned inflammatory factors incite the migration of inflammatory cells, predominantly T cells, into the renal interstitium, blood vessels, and glomeruli. This results in an excessive accumulation of collagen and extracellular matrix, which ultimately culminates in renal tissue fibrosis. Spironolactone has a direct inhibitory effect on T cell activation based on the inhibition of NF-κB activity [22]. In the study conducted by Sandor et al. [47], low-dose MRA has also been demonstrated to directly reduce the effects of renal profibrotic factors (TGFβ1, PDGF, CTGF/CCN2, etc.) and the production of extracellular matrix (ECM) by fibroblasts. The substantial accumulation of extracellular matrix (ECM) represents a significant pathological foundation for the development of glomerulosclerosis [48]. Studies show pronounced immunosenescence in CKD patients, characterized by T/B cell dysregulation/accumulation and elevated pro-inflammatory cytokines (e.g., IL-17/IL-23) [3].

## Comparison of steroidal and nonsteroidal MRA antagonists

### Differences in pharmacological mechanisms

Spironolactone, a classic mineralocorticoid receptor antagonist developed 50 years ago for hypertension, remains in use but causes nonselective effects (e.g., the male breast gland, hyperkalemia, and renal impairment). The more selective MR antagonist eplerenone was later developed, but shows reduced efficacy and a short 3-h half-life [49, 50]. Both drugs carry significant hyperkalemia risks.

A number of drugs are currently in development (CS-3150, PF-03882845, DSR-71167), with finerenone representing the most advanced. Its chemical structure was derived from that of dihydropyridine channel blockers, without any activity against L-type calcium channels [51]. Finerenone has a considerably shorter half-life than steroidal MRAs and lacks active metabolites [52]. Additionally, it exhibits greater MR selectivity than spironolactone and a higher receptor binding affinity than eplerenone, and demonstrates a reduction in serum potassium elevation, a lower incidence of hyperkalemia, and a lower incidence of renal impairment in patients with heart failure and chronic kidney disease [53, 54].

### Preclinical research

MR antagonists (MRAs) exhibit significant anti-inflammatory effects by reducing immune cell infiltration. In myeloid-specific MR knockout (MyMRKO) mice, eplerenone treatment markedly decreased neutrophil, CD4 + /CD8 + T cell, and macrophage accumulation, along with inflammatory mediators (TNF-α, matrix metalloproteinase12, inducible nitric oxide synthase) [55]. TNF-α, a key pro-inflammatory cytokine in hypertensive end-organ damage, is suppressed by MRAs. Eplerenone also downregulates renal leukocyte adhesion molecules and pro-inflammatory genes, particularly inhibiting macrophage recruitment and M2 phenotype polarization [56–58]. Mechanistically, MRAs block MR-PPARγ crosstalk in macrophages, promoting an M2 anti-inflammatory state [58]. In vitro studies further confirm MR’s direct modulation of CD4 + T cell activation.

Comparison with the steroidal MR antagonist, eplerenone, finerenone is more effective at reducing the production and release of pro-inflammatory cytokines and can effectively reduce macrophage infiltration and activation, in preclinical rat disease models, and significantly reduces immune cell activity and infiltration in cardiorenal tissue [53]. Kidney injury can arouse the secretion of numerous inflammatory cells, which in turn secrete a variety of growth factors, including TGF-β, EGF, and FGF-2. These growth factors stimulate epithelial–mesenchymal transition (EMT). During the process of fibrosis, some subpopulations of these fibroblasts express αSMA and synthesize a substantial quantity of extracellular matrix proteins, leading to fibrosis of renal tissue, particularly affecting renal tubular epithelial cells [59]. In diabetic mice fed a high-salt diet, the novel nonsteroidal MRA esaxerenone demonstrated efficacy in inhibiting renal inflammation and renal fibrosis by selectively blocking MR, as evidenced by its superior efficacy compared to spironolactone [60].

Finerenone protects against functional and structural renal and cardiac damage in rats at doses that do not reduce systemic blood pressure [53]. Kolkhof et al. observed that [C]-labeled finerenone was distributed uniformly in the hearts and kidneys of rats using quantitative whole-body autoradiography. In comparison to eplerenone, it proved to be more efficacious in the reduction of myocardial hypertrophy, plasma pro-brain natriuretic peptide hormone levels, and proteinuria.

The new nonsteroidal MRA has good benefits in the prevention of acute kidney injury following ischemia–reperfusion and its subsequent transformation to chronicity. Lattenist et al. demonstrated that finerenone can prevent the increase of oxidative stress and ET-B sulfenic acid modification in the kidney after ischemia [61]. The MR antagonism of finerenone can reduce the mRNA levels of lipoprotein-related tubular injury markers, including kidney injury molecule-1 and neutrophil gelatinase. Moreover, this mechanism has been shown to prevent the development of acute kidney injury in models of ischemia–reperfusion (IR).

## Conclusion and future outlook

Notably, the nonsteroidal MRA finerenone effectively reduces inflammation, proteinuria, and renal damage without significant hyperkalemia risk. Studies demonstrate its superior cardiorenal benefits and safety compared to older MRAs. By modulating T cell function, inhibiting pro-inflammatory cytokines, and reducing oxidative stress, finerenone shows promising cardiorenal protective effects.

The data will facilitate the identification of new biomarkers and therapeutic targets. Furthermore, the reduction of inflammatory factor production, the specific pathway knockout, and the inhibition of immune cell subtype transformation may represent a novel direction for the development of treatments.

This review highlights the key roles of both steroidal and nonsteroidal MRAs in immune pathways during renal injury progression. MRAs provide significant protection by counteracting harmful mineralocorticoid effects on kidneys and heart. They reduce renal inflammation, decrease pro-inflammatory factors, and slow tissue fibrosis. Additionally, MRAs improve T cell function, modulate immune responses, and limit immune cell infiltration, as demonstrated in diabetic and high-salt diet kidney injury models.

There are notable distinctions in the molecular and pharmacological characteristics between steroidal and nonsteroidal MRAs. In particular, the new nonsteroidal MRA finerenone has been demonstrated to effectively reduce the inflammatory response, reduce proteinuria and renal structural damage without significantly increasing the risk of hyperkalemia. The results of these studies illustrate that the drug has notable benefits for the kidneys and heart, while also demonstrating a favorable safety profile in comparison to older MRA medications. MRA shows promise in improving cardiorenal function by modulating T cell function, inhibiting the release of pro-inflammatory cytokines, and alleviating oxidative stress.

Emerging research directions in CKD treatment should focus on elucidating the blood pressure-independent cardiorenal protective mechanisms of MRAs, as demonstrated in FIDELIO-DKD, including their potential effects on neutrophils and NK cells observed in angiotensin II-induced hypertension models [62, 63]. Therapeutic strategies could target multiple inflammatory pathways (IL-4R, MAPK-JNK, IL-17/23 axis, and NF-κB downstream factors) to reduce oxidative stress and macrophage infiltration, while novel approaches combining MRAs with IL-17 inhibitors or senolytics may simultaneously address immunosenescence and cellular senescence. Additionally, modulation of immune cell subtypes and selective channel inhibition represent promising treatment avenues, though further studies are needed to validate these mechanisms and develop optimized therapeutic regimens for CKD management.

## Data Availability

No datasets were generated or analysed during the current study.

## Abbreviations

CKD: Chronic kidney disease
MR: Mineralocorticoid receptors
GFR: Glomerular filtration rate
MRAs: Mineralocorticoid receptor antagonists
MAPK: Mitogen-activated protein kinase
VEGFR3: Vascular endothelial growth factor receptor 3
TFEB: Transcription factor EB
PRKN: Parkin RBR E3 ubiquitin protein ligase
NFAT-1: Nuclear factor of activated T cells 1
AP1: Activator protein-1
Treg: T regulatory cell
SKG1: Serine/threonine protein inhibitor 1
NCC: Sodium-chloride cotransporter
ENaC: Sodium epithelial channel
NGAL: Neutrophil gelatinase-associated lipocalin
PPAR-γ: Peroxisome proliferator-activated receptor γ
NF-κB: Nuclear factor kappa B; CT-1, cardiotrophin-1
TLR4: Toll-like receptor 4; ECM, extracellular matrix
TMRKO: T cell MR knockout
EMT: Epithelial–mesenchymal transition
T2DM: Type 2 diabetes mellitus
UACR: Urinary albumin-to-creatinine ratio
NK cell: Natural killer cell

## References

[1] Vaziri ND, Pahl MV, Crum A, Norris K (2012) Effect of uremia on structure and function of immune system. J Ren Nutr 22:149–156. 10.1053/j.jrn.2011.10.020 22200433 10.1053/j.jrn.2011.10.020 PMC3246616

[2] Nikolich-Žugich J (2018) The twilight of immunity: emerging concepts in aging of the immune system. Nat Immunol 19:10–19. 10.1038/s41590-017-0006-x 29242543 10.1038/s41590-017-0006-x

[3] Tang Y, Jiang J, Zhao Y, Du D (2024) Aging and chronic kidney disease: epidemiology, therapy, management and the role of immunity. Clin Kidney J 17:sfae235. 10.1093/ckj/sfae235 40034487 10.1093/ckj/sfae235 PMC11873799

[4] Brown NJ (2013) Contribution of aldosterone to cardiovascular and renal inflammation and fibrosis. Nat Rev Nephrol 9:459–469. 10.1038/nrneph.2013.110 23774812 10.1038/nrneph.2013.110 PMC3922409

[5] Bauersachs J, Jaisser F, Toto R (2015) Mineralocorticoid receptor activation and mineralocorticoid receptor antagonist treatment in cardiac and renal diseases. Hypertension 65:257–263. 10.1161/HYPERTENSIONAHA.114.04488 25368026 10.1161/HYPERTENSIONAHA.114.04488

[6] Funder JW (2005) Mineralocorticoid receptors: distribution and activation. Heart Fail Rev 10:15–22. 10.1007/s10741-005-2344-2 15947887 10.1007/s10741-005-2344-2

[7] Barrera-Chimal J, Girerd S, Jaisser F (2019) Mineralocorticoid receptor antagonists and kidney diseases: pathophysiological basis. Kidney Int 96:302–319. 10.1016/j.kint.2019.02.030 31133455 10.1016/j.kint.2019.02.030

[8] Kowalski J, Deng L, Suennen C, Koca D, Meral D, Bode C, Hein L, Lother A (2021) Eplerenone improves pulmonary vascular remodeling and hypertension by inhibition of the mineralocorticoid receptor in endothelial cells. Hypertension 78:456–465. 10.1161/HYPERTENSIONAHA.120.16196 33966455 10.1161/HYPERTENSIONAHA.120.16196

[9] Kasal DA, Barhoumi T, Li MW, Yamamoto N, Zdanovich E, Rehman A, Neves MF, Laurant P, Paradis P, Schiffrin EL (2012) T regulatory lymphocytes prevent aldosterone-induced vascular injury. Hypertension 59:324–330. 10.1161/HYPERTENSIONAHA.111.181123 22146512 10.1161/HYPERTENSIONAHA.111.181123

[10] Lu Y, Ku E, Campese VM (2010) Aldosterone in the pathogenesis of chronic kidney disease and proteinuria. Curr Hypertens Rep 12:303–306. 10.1007/s11906-010-0116-4 20596805 10.1007/s11906-010-0116-4

[11] Barrera-Chimal J, Estrela GR, Lechner SM, Giraud S, El Moghrabi S, Kaaki S, Kolkhof P, Hauet T, Jaisser F (2018) The myeloid mineralocorticoid receptor controls inflammatory and fibrotic responses after renal injury via macrophage interleukin-4 receptor signaling. Kidney Int 93:1344–1355. 10.1016/j.kint.2017.12.016 29548765 10.1016/j.kint.2017.12.016

[12] Doi T, Doi S, Nakashima A, Ueno T, Yokoyama Y, Kohno N, Masaki T (2014) Mizoribine ameliorates renal injury and hypertension along with the attenuation of renal caspase-1 expression in aldosterone-salt-treated rats. PLoS ONE 9:e93513. 10.1371/journal.pone.0093513 24695748 10.1371/journal.pone.0093513 PMC3973594

[13] Kang K, Reilly SM, Karabacak V, Gangl MR, Fitzgerald K, Hatano B, Lee C-H (2008) Adipocyte-derived Th2 cytokines and myeloid PPARdelta regulate macrophage polarization and insulin sensitivity. Cell Metab 7:485–495. 10.1016/j.cmet.2008.04.002 18522830 10.1016/j.cmet.2008.04.002 PMC2586840

[14] Odegaard JI, Ricardo-Gonzalez RR, Goforth MH, Morel CR, Subramanian V, Mukundan L, Red Eagle A, Vats D, Brombacher F, Ferrante AW, Chawla A (2007) Macrophage-specific PPARgamma controls alternative activation and improves insulin resistance. Nature 447:1116–1120. 10.1038/nature05894 17515919 10.1038/nature05894 PMC2587297

[15] Sun J-Y, Li C, Shen Z-X, Zhang W-C, Ai T-J, Du L-J, Zhang Y-Y, Yao G-F, Liu Y, Sun S, Naray-Fejes-Toth A, Fejes-Toth G, Peng Y, Chen M, Liu X, Tao J, Zhou B, Yu Y, Guo F, Du J, Duan S-Z (2016) Mineralocorticoid receptor deficiency in macrophages inhibits neointimal hyperplasia and suppresses macrophage inflammation through SGK1-AP1/NF-κB pathways. Arterioscler Thromb Vasc Biol 36:874–885. 10.1161/ATVBAHA.115.307031 26966277 10.1161/ATVBAHA.115.307031

[16] Ong GSY, Cole TJ, Tesch GH, Morgan J, Dowling JK, Mansell A, Fuller PJ, Young MJ (2020) Novel mineralocorticoid receptor mechanisms regulate cardiac tissue inflammation in male mice. J Endocrinol 246:123–134. 10.1530/JOE-20-0161 32464598 10.1530/JOE-20-0161

[17] Xu S, Wang D, Tan L, Lu J (2024) The role of NLRP3 inflammasome in type 2 inflammation related diseases. Autoimmunity 57:2310269. 10.1080/08916934.2024.2310269 38332696 10.1080/08916934.2024.2310269

[18] Cooper SA, Whaley-Connell A, Habibi J, Wei Y, Lastra G, Manrique C, Stas S, Sowers JR (2007) Renin-angiotensin-aldosterone system and oxidative stress in cardiovascular insulin resistance. Am J Physiol Heart Circ Physiol 293:H2009-2023. 10.1152/ajpheart.00522.2007 17586614 10.1152/ajpheart.00522.2007

[19] Wu Q, Meng W, Zhu B, Chen X, Fu J, Zhao C, Liu G, Luo X, Lv Y, Zhao W, Wang F, Hu S, Zhang S (2023) VEGFC ameliorates salt-sensitive hypertension and hypertensive nephropathy by inhibiting NLRP3 inflammasome via activating VEGFR3-AMPK dependent autophagy pathway. Cell Mol Life Sci 80:327. 10.1007/s00018-023-04978-3 37837447 10.1007/s00018-023-04978-3 PMC11072217

[20] Ferreira NS, Tostes RC, Paradis P, Schiffrin EL (2021) Aldosterone, inflammation, immune system, and hypertension. Am J Hypertens 34:15–27. 10.1093/ajh/hpaa137 32820797 10.1093/ajh/hpaa137 PMC7891246

[21] Sun X-N, Li C, Liu Y, Du L-J, Zeng M-R, Zheng X-J, Zhang W-C, Liu Y, Zhu M, Kong D, Zhou L, Lu L, Shen Z-X, Yi Y, Du L, Qin M, Liu X, Hua Z, Sun S, Yin H, Zhou B, Yu Y, Zhang Z, Duan S-Z (2017) T-cell mineralocorticoid receptor controls blood pressure by regulating interferon-gamma. Circ Res 120:1584–1597. 10.1161/CIRCRESAHA.116.310480 28298295 10.1161/CIRCRESAHA.116.310480

[22] Herrada AA, Contreras FJ, Marini NP, Amador CA, González PA, Cortés CM, Riedel CA, Carvajal CA, Figueroa F, Michea LF, Fardella CE, Kalergis AM (2010) Aldosterone promotes autoimmune damage by enhancing Th17-mediated immunity. J Immunol 184:191–202. 10.4049/jimmunol.0802886 19949098 10.4049/jimmunol.0802886

[23] Dourado TMH, Assis VO, Awata WMC, de Mello MMB, Cárnio EC, Castro MM, Tirapelli CR (2023) Mineralocorticoid receptors contribute to ethanol-induced vascular hypercontractility through reactive oxygen species generation and up-regulation of cyclooxygenase 2. Eur J Pharmacol 949:175723. 10.1016/j.ejphar.2023.175723 37059378 10.1016/j.ejphar.2023.175723

[24] Dourado TMH, Nascimento DC, Rosa MH, Assis VO, Pimenta GF, Alves-Filho JC, Tirapelli CR (2024) Mineralocorticoid receptor antagonism partially prevents dysfunction of T cell maturation in rats chronically treated with ethanol. Naunyn Schmiedebergs Arch Pharmacol. 10.1007/s00210-024-03382-3 39172146 10.1007/s00210-024-03382-3

[25] Li C, Sun X-N, Zeng M-R, Zheng X-J, Zhang Y-Y, Wan Q, Zhang W-C, Shi C, Du L-J, Ai T-J, Liu Y, Liu Y, Du L-L, Yi Y, Yu Y, Duan S-Z (2017) Mineralocorticoid receptor deficiency in T cells attenuates pressure overload-induced cardiac hypertrophy and dysfunction through modulating T-cell activation. Hypertension 70:137–147. 10.1161/HYPERTENSIONAHA.117.09070 28559389 10.1161/HYPERTENSIONAHA.117.09070

[26] Amador CA, Barrientos V, Peña J, Herrada AA, González M, Valdés S, Carrasco L, Alzamora R, Figueroa F, Kalergis AM, Michea L (2014) Spironolactone decreases DOCA-salt-induced organ damage by blocking the activation of T helper 17 and the downregulation of regulatory T lymphocytes. Hypertension 63:797–803. 10.1161/HYPERTENSIONAHA.113.02883 24420551 10.1161/HYPERTENSIONAHA.113.02883

[27] Krebs CF, Lange S, Niemann G, Rosendahl A, Lehners A, Meyer-Schwesinger C, Stahl RAK, Benndorf RA, Velden J, Paust H-J, Panzer U, Ehmke H, Wenzel UO (2014) Deficiency of the interleukin 17/23 axis accelerates renal injury in mice with deoxycorticosterone acetate+angiotensin ii-induced hypertension. Hypertension 63:565–571. 10.1161/HYPERTENSIONAHA.113.02620 24366079 10.1161/HYPERTENSIONAHA.113.02620

[28] Zeng X, Wei Y-L, Huang J, Newell EW, Yu H, Kidd BA, Kuhns MS, Waters RW, Davis MM, Weaver CT, Chien Y (2012) γδ T cells recognize a microbial encoded B cell antigen to initiate a rapid antigen-specific interleukin-17 response. Immunity 37:524–534. 10.1016/j.immuni.2012.06.011 22960222 10.1016/j.immuni.2012.06.011 PMC3495981

[29] Luettges K, Bode M, Diemer JN, Schwanbeck J, Wirth EK, Klopfleisch R, Kappert K, Thiele A, Ritter D, Foryst-Ludwig A, Kolkhof P, Wenzel UO, Kintscher U (2022) Finerenone reduces renal RORγt γδ T cells and protects against cardiorenal damage. Am J Nephrol 53:552–564. 10.1159/000524940 35675794 10.1159/000524940

[30] Teixeira LK, Fonseca BPF, Vieira-de-Abreu A, Barboza BA, Robbs BK, Bozza PT, Viola JPB (2005) IFN-gamma production by CD8+ T cells depends on NFAT1 transcription factor and regulates Th differentiation. J Immunol 175:5931–5939. 10.4049/jimmunol.175.9.5931 16237086 10.4049/jimmunol.175.9.5931

[31] Trott DW, Thabet SR, Kirabo A, Saleh MA, Itani H, Norlander AE, Wu J, Goldstein A, Arendshorst WJ, Madhur MS, Chen W, Li C-I, Shyr Y, Harrison DG (2014) Oligoclonal CD8+ T cells play a critical role in the development of hypertension. Hypertension 64:1108–1115. 10.1161/HYPERTENSIONAHA.114.04147 25259750 10.1161/HYPERTENSIONAHA.114.04147 PMC4191997

[32] Schmieder RE (2010) End organ damage in hypertension. Dtsch Arztebl Int 107:866–873. 10.3238/arztebl.2010.0866 21191547 10.3238/arztebl.2010.0866 PMC3011179

[33] Ellison DH (2013) Ubiquitylation and the pathogenesis of hypertension. J Clin Invest 123:546–548. 10.1172/JCI66882 23348731 10.1172/JCI66882 PMC3561829

[34] Berger RCM, Vassallo PF, Crajoinas RD, Oliveira ML, Martins FL, Nogueira BV, Motta-Santos D, Araújo IB, Forechi L, Girardi ACC, Santos RAS, Mill JG (2015) Renal effects and underlying molecular mechanisms of long-term salt content diets in spontaneously hypertensive rats. PLoS ONE 10:e0141288. 10.1371/journal.pone.0141288 26495970 10.1371/journal.pone.0141288 PMC4619858

[35] Yu HC, Burrell LM, Black MJ, Wu LL, Dilley RJ, Cooper ME, Johnston CI (1998) Salt induces myocardial and renal fibrosis in normotensive and hypertensive rats. Circulation 98:2621–2628. 10.1161/01.cir.98.23.2621 9843472 10.1161/01.cir.98.23.2621

[36] McMaster WG, Kirabo A, Madhur MS, Harrison DG (2015) Inflammation, immunity, and hypertensive end-organ damage. Circ Res 116:1022–1033. 10.1161/CIRCRESAHA.116.303697 25767287 10.1161/CIRCRESAHA.116.303697 PMC4535695

[37] Norlander AE, Saleh MA, Pandey AK, Itani HA, Wu J, Xiao L, Kang J, Dale BL, Goleva SB, Laroumanie F, Du L, Harrison DG, Madhur MS (2017) A salt-sensing kinase in T lymphocytes, SGK1, drives hypertension and hypertensive end-organ damage. JCI Insight 2(e92801):92801. 10.1172/jci.insight.92801 28679951 10.1172/jci.insight.92801 PMC5499362

[38] Maaliki D, Itani M, Jarrah H, El-Mallah C, Ismail D, El Atie YE, Obeid O, Jaffa MA, Itani HA (2024) Dietary high salt intake exacerbates SGK1-mediated T cell pathogenicity in L-NAME/high salt-induced hypertension. Int J Mol Sci 25:4402. 10.3390/ijms25084402 38673987 10.3390/ijms25084402 PMC11050194

[39] Buonafine M, Martínez-Martínez E, Amador C, Gravez B, Ibarrola J, Fernández-Celis A, El Moghrabi S, Rossignol P, López-Andrés N, Jaisser F (2018) Neutrophil gelatinase-associated lipocalin from immune cells is mandatory for aldosterone-induced cardiac remodeling and inflammation. J Mol Cell Cardiol 115:32–38. 10.1016/j.yjmcc.2017.12.011 29289651 10.1016/j.yjmcc.2017.12.011

[40] Bonnard B, Ibarrola J, Lima-Posada I, Fernández-Celis A, Durand M, Genty M, Lopez-Andrés N, Jaisser F (2022) Neutrophil gelatinase-associated lipocalin from macrophages plays a critical role in renal fibrosis via the CCL5 (Chemokine Ligand 5)-Th2 cells-IL4 (Interleukin 4) pathway. Hypertension 79:352–364. 10.1161/HYPERTENSIONAHA.121.17712 34794340 10.1161/HYPERTENSIONAHA.121.17712

[41] Duni A, Liakopoulos V, Roumeliotis S, Peschos D, Dounousi E (2019) Oxidative stress in the pathogenesis and evolution of chronic kidney disease: untangling Ariadne’s thread. Int J Mol Sci 20:3711. 10.3390/ijms20153711 31362427 10.3390/ijms20153711 PMC6695865

[42] Remuzzi G, Bertani T (1998) Pathophysiology of progressive nephropathies. N Engl J Med 339:1448–1456. 10.1056/NEJM199811123392007 9811921 10.1056/NEJM199811123392007

[43] Toll-like receptor 4 contributes to vascular remodelling and endothelial dysfunction in angiotensin II-induced hypertension - PubMed. https://pubmed.ncbi.nlm.nih.gov/25712370/. Accessed 20 Mar 2024 10.1111/bph.13117 PMC4459031 25712370

[44] López-Andrés N, Martin-Fernandez B, Rossignol P, Zannad F, Lahera V, Fortuno MA, Cachofeiro V, Díez J (2011) A role for cardiotrophin-1 in myocardial remodeling induced by aldosterone. Am J Physiol Heart Circ Physiol 301:H2372-2382. 10.1152/ajpheart.00283.2011 21926338 10.1152/ajpheart.00283.2011

[45] Miana M, de Las HN, Rodriguez C, Sanz-Rosa D, Martin-Fernandez B, Mezzano S, Lahera V, Martinez-Gonzalez J, Cachofeiro V (2011) Effect of eplerenone on hypertension-associated renal damage in rats: potential role of peroxisome proliferator activated receptor gamma (PPAR-γ). J Physiol Pharmacol 62:87–94 21451213

[46] Echem C, Bomfim GF, Ceravolo GS, Oliveira MA, Santos-Eichler RA, Bechara LR, Veras MM, Saldiva PHN, Ferreira JC, Akamine EH, Fortes ZB, Dantas AP, de Carvalho MHC (2015) Anti-toll like receptor 4 (TLR4) therapy diminishes cardiac remodeling regardless of changes in blood pressure in spontaneously hypertensive rats (SHR). Int J Cardiol 187:243–245. 10.1016/j.ijcard.2015.03.190 25838224 10.1016/j.ijcard.2015.03.190

[47] Koszegi S, Molnar A, Lenart L, Hodrea J, Balogh DB, Lakat T, Szkibinszkij E, Hosszu A, Sparding N, Genovese F, Wagner L, Vannay A, Szabo AJ, Fekete A (2019) RAAS inhibitors directly reduce diabetes-induced renal fibrosis via growth factor inhibition. J Physiol 597:193–209. 10.1113/JP277002 30324679 10.1113/JP277002 PMC6312411

[48] Li L, Fu H, Liu Y (2022) The fibrogenic niche in kidney fibrosis: components and mechanisms. Nat Rev Nephrol 18:545–557. 10.1038/s41581-022-00590-z 35788561 10.1038/s41581-022-00590-z

[49] de Gasparo M, Joss U, Ramjoué HP, Whitebread SE, Haenni H, Schenkel L, Kraehenbuehl C, Biollaz M, Grob J, Schmidlin J (1987) Three new epoxy-spirolactone derivatives: characterization in vivo and in vitro. J Pharmacol Exp Ther 240:650–656 2949071

[50] Cook CS, Berry LM, Bible RH, Hribar JD, Hajdu E, Liu NW (2003) Pharmacokinetics and metabolism of [14C]eplerenone after oral administration to humans. Drug Metab Dispos 31:1448–1455. 10.1124/dmd.31.11.1448 14570778 10.1124/dmd.31.11.1448

[51] Bärfacker L, Kuhl A, Hillisch A, Grosser R, Figueroa-Pérez S, Heckroth H, Nitsche A, Ergüden J-K, Gielen-Haertwig H, Schlemmer K-H, Mittendorf J, Paulsen H, Platzek J, Kolkhof P (2012) Discovery of BAY 94–8862: a nonsteroidal antagonist of the mineralocorticoid receptor for the treatment of cardiorenal diseases. ChemMedChem 7:1385–1403. 10.1002/cmdc.201200081 22791416 10.1002/cmdc.201200081

[52] Agarwal R, Kolkhof P, Bakris G, Bauersachs J, Haller H, Wada T, Zannad F (2021) Steroidal and non-steroidal mineralocorticoid receptor antagonists in cardiorenal medicine. Eur Heart J 42:152–161. 10.1093/eurheartj/ehaa736 33099609 10.1093/eurheartj/ehaa736 PMC7813624

[53] Kolkhof P, Delbeck M, Kretschmer A, Steinke W, Hartmann E, Bärfacker L, Eitner F, Albrecht-Küpper B, Schäfer S (2014) Finerenone, a novel selective nonsteroidal mineralocorticoid receptor antagonist protects from rat cardiorenal injury. J Cardiovasc Pharmacol 64:69–78. 10.1097/FJC.0000000000000091 24621652 10.1097/FJC.0000000000000091

[54] Pitt B, Kober L, Ponikowski P, Gheorghiade M, Filippatos G, Krum H, Nowack C, Kolkhof P, Kim S-Y, Zannad F (2013) Safety and tolerability of the novel non-steroidal mineralocorticoid receptor antagonist BAY 94–8862 in patients with chronic heart failure and mild or moderate chronic kidney disease: a randomized, double-blind trial. Eur Heart J 34:2453–2463. 10.1093/eurheartj/eht187 23713082 10.1093/eurheartj/eht187 PMC3743070

[55] Huang LL, Nikolic-Paterson DJ, Han Y, Ozols E, Ma FY, Young MJ, Tesch GH (2014) Myeloid mineralocorticoid receptor activation contributes to progressive kidney disease. J Am Soc Nephrol 25:2231–2240. 10.1681/ASN.2012111094 24700867 10.1681/ASN.2012111094 PMC4178428

[56] Han Y, Ma FY, Tesch GH, Manthey CL, Nikolic-Paterson DJ (2013) Role of macrophages in the fibrotic phase of rat crescentic glomerulonephritis. Am J Physiol Renal Physiol 304:F1043-1053. 10.1152/ajprenal.00389.2012 23408165 10.1152/ajprenal.00389.2012

[57] Kobayashi N, Hara K, Tojo A, Onozato ML, Honda T, Yoshida K, Mita S, Nakano S, Tsubokou Y, Matsuoka H (2005) Eplerenone shows renoprotective effect by reducing LOX-1-mediated adhesion molecule, PKCepsilon-MAPK-p90RSK, and Rho-kinase pathway. Hypertension 45:538–544. 10.1161/01.HYP.0000157408.43807.5a 15710785 10.1161/01.HYP.0000157408.43807.5a

[58] Usher MG, Duan SZ, Ivaschenko CY, Frieler RA, Berger S, Schütz G, Lumeng CN, Mortensen RM (2010) Myeloid mineralocorticoid receptor controls macrophage polarization and cardiovascular hypertrophy and remodeling in mice. J Clin Invest 120:3350–3364. 10.1172/JCI41080 20697155 10.1172/JCI41080 PMC2929712

[59] Kalluri R, Neilson EG (2003) Epithelial-mesenchymal transition and its implications for fibrosis. J Clin Invest 112:1776–1784. 10.1172/JCI20530 14679171 10.1172/JCI20530 PMC297008

[60] Bhuiyan AS, Rafiq K, Kobara H, Masaki T, Nakano D, Nishiyama A (2019) Effect of a novel nonsteroidal selective mineralocorticoid receptor antagonist, esaxerenone (CS-3150), on blood pressure and renal injury in high salt-treated type 2 diabetic mice. Hypertens Res 42:892–902. 10.1038/s41440-019-0211-0 30664703 10.1038/s41440-019-0211-0

[61] Lattenist L, Lechner SM, Messaoudi S, Le Mercier A, El Moghrabi S, Prince S, Bobadilla NA, Kolkhof P, Jaisser F, Barrera-Chimal J (2017) Nonsteroidal mineralocorticoid receptor antagonist finerenone protects against acute kidney injury-mediated chronic kidney disease. Hypertension 69:870–878. 10.1161/HYPERTENSIONAHA.116.08526 28320854 10.1161/HYPERTENSIONAHA.116.08526

[62] Sanders PW, Wang P-X (2002) Activation of the Fas/Fas ligand pathway in hypertensive renal disease in Dahl/Rapp rats. BMC Nephrol 3:1. 10.1186/1471-2369-3-1 11818026 10.1186/1471-2369-3-1 PMC64784

[63] Travis OK, White D, Pierce WA, Ge Y, Stubbs CY, Spradley FT, Williams JM, Cornelius DC (2019) Chronic infusion of interleukin-17 promotes hypertension, activation of cytolytic natural killer cells, and vascular dysfunction in pregnant rats. Physiol Rep 7:e14038. 10.14814/phy2.14038 30963715 10.14814/phy2.14038 PMC6453821

